# Randomized Trial of Fetal Surgery for Severe Left Diaphragmatic Hernia

**DOI:** 10.1056/NEJMoa2027030

**Published:** 2021-06-08

**Authors:** J.A. Deprest, K.H. Nicolaides, A. Benachi, E. Gratacos, G. Ryan, N. Persico, H. Sago, A. Johnson, M. Wielgoś, C. Berg, B. Van Calster, F.M. Russo, for the TOTAL Trial for Severe Hypoplasia Investigators

**Affiliations:** the Department of Obstetrics and Gynecology, KU Leuven, Academic Department of Development and Regeneration, Biomedical Sciences, University Hospitals KU Leuven, Leuven, Belgium, Institute for Women’s Health, University College London Hospital in London; King’s College Hospital, the Institute for Women’s Health, University College London Hospital in London; Hospital Antoine-Béclère, Université Paris-Saclay, Clamart, France; Hospital Clinic and Sant Joan de Déu, Barcelona; Mount Sinai Hospital, Toronto; Hospital Maggiore Policlinico, Milan; the National Center for Child Health and Development, Tokyo; Children’s Memorial Hermann Hospital, Houston; the Medical University of Warsaw, Warsaw, Poland; University Hospital Bonn, Bonn, Germany; Academic Department of Development and Regeneration, Biomedical Sciences, University Hospitals KU Leuven, Leuven, Belgium; the Department of Obstetrics and Gynecology, KU Leuven, Academic Department of Development and Regeneration, Biomedical Sciences, University Hospitals KU Leuven, Leuven, Belgium

## Abstract

**Background:**

Observational studies have shown that fetoscopic endoluminal tracheal occlusion (FETO) has been associated with increased survival among infants with severe pulmonary hypoplasia due to isolated congenital diaphragmatic hernia on the left side, but data from randomized trials are lacking.

**Methods:**

In this open-label trial conducted at centers with experience in FETO and other types of prenatal surgery, we randomly assigned, in a 1:1 ratio, women carrying singleton fetuses with severe isolated congenital diaphragmatic hernia on the left side to FETO at 27 to 29 weeks of gestation or expectant care. Both treatments were followed by standardized postnatal care. The primary outcome was infant survival to discharge from the neonatal intensive care unit. We used a group-sequential design with five prespecified interim analyses for superiority, with a maximum sample size of 116 women.

**Results:**

The trial was stopped early for efficacy after the third interim analysis. In an intention-to-treat analysis that included 80 women, 40% of infants (16 of 40) in the FETO group survived to discharge, as compared with 15% (6 of 40) in the expectant care group (relative risk, 2.67; 95% confidence interval [CI], 1.22 to 6.11; two-sided P=0.009). Survival to 6 months of age was identical to the survival to discharge (relative risk, 2.67; 95% CI, 1.22 to 6.11). The incidence of preterm, prelabor rupture of membranes was higher among women in the FETO group than among those in the expectant care group (47% vs. 11%; relative risk, 4.51; 95% CI, 1.83 to 11.9), as was the incidence of preterm birth (75% vs. 29%; relative risk, 2.59; 95% CI, 1.59 to 4.52). One neonatal death occurred after emergency delivery for placental laceration from fetoscopic balloon removal, and one neonatal death occurred because of failed balloon removal. In an analysis that included 11 additional participants with data that were available after the trial was stopped, survival to discharge was 36% among infants in the FETO group and 14% among those in the expectant care group (relative risk, 2.65; 95% CI, 1.21 to 6.09).

**Conclusions:**

In fetuses with isolated severe congenital diaphragmatic hernia on the left side, FETO performed at 27 to 29 weeks of gestation resulted in a significant benefit over expectant care with respect to survival to discharge, and this benefit was sustained to 6 months of age. FETO increased the risks of preterm, prelabor rupture of membranes and preterm birth. (Funded by the European Commission and others; TOTAL ClinicalTrials.gov number, NCT01240057.)

The Prevalence of Congenital Diaphragmatic hernia is approximately 1 in 4000 births, and in 85% of cases, the defect is on the left side.^1,2^ In congenital diaphragmatic hernia, intrathoracic herniation of the abdominal viscera impairs normal airway and pulmonary vascular development.^3^ Consequently, the condition is associated with a high risk of neonatal death due to respiratory failure and pulmonary hypertension.^4^ Infants who survive may have serious health complications, including gastrointestinal and respiratory problems, orthopedic deformations, and neurodevelopmental delay. Lifelong multidisciplinary follow-up for early diagnosis and management of complications is necessary in children with congenital diaphragmatic hernia.^5^ In the United States, the costs of postnatal care for patients with congenital diaphragmatic hernia exceed $250 million per year, making it the most costly noncardiac birth defect.^6^

In fetuses with congenital diaphragmatic hernia, prenatal assessment to determine the most likely postnatal survival outcome is informed by the presence of other major defects and chromosomal abnormalities, the measurement of lung size, and determination of whether there is intrathoracic herniation of the liver on ultrasonography or magnetic resonance imaging. Lung size is generally assessed on the basis of the ratio of the contralateral lung area (measured on a two-dimensional ultrasonographic scan showing the standard four-chamber view of the heart) to the head circumference.^7^ Because these measurements vary with gestational age, lung size is better expressed as the quotient of the observed-to-expected lung-to-head ratios (i.e., the ratio of the observed lung area to head circumference [measured on ultrasonography] divided by the ratio of that which would be expected in a healthy fetus of the same gestational age).^8^ Fetuses with a quotient of observed-to-expected lung-to-head ratios of less than 25.0% are referred to as having severe pulmonary hypoplasia, and their chance of survival is less than 25%.^8^ The quotient of the observed-to-expected lung-to-head ratios is also predictive of early neonatal complications such as the extended use of ventilation and the need for supplemental oxygen, as well as of the time to full enteral feeding.^9–11^

Fetal lung growth is stimulated by tracheal obstruction, as has been observed in congenital high airway obstruction, as well as after experimental fetal tracheal ligation.^12–15^ Experiments have also shown that maturation is stimulated by prenatal reversal of the occlusion and administration of glucocortocoids.^16,17^ Clinical tracheal occlusion was first achieved by applying an external clip while the mother was under general anesthesia.^18^ We developed a fetoscopic endoluminal tracheal occlusion (FETO) technique involving endoluminal insertion of an inflatable balloon, with removal of the balloon a few weeks later; insertion and removal can be performed while the mother is under local anesthesia.^19^ In a study involving 210 fetuses with severe hypoplasia due to isolated congenital diaphragmatic hernia, the use of FETO (with balloon insertion at approximately 28 weeks of gestation and removal at 34 weeks of gestation) appeared to have an acceptable safety profile in the mother. As compared with historical controls, neonatal survival among these fetuses was higher than among historical controls (49% vs. 24%) and the incidence of early neonatal respiratory complications was lower, but the risk of premature birth was higher.^11,20^ We designed the Tracheal Occlusion to Accelerate Lung Growth (TOTAL) trial (www.totaltrial.eu) to test the hypothesis that in fetuses with severe pulmonary hypoplasia due to isolated congenital diaphragmatic hernia on the left side, the use of FETO, as compared with expectant prenatal care.^21,22^ (with both treatments followed by standardized postnatal care), may increase postnatal survival. A companion article now published in the *Journal* describes a randomized trial involving fetuses with isolated congenital diaphragmatic hernia and moderate pulmonary hypoplasia on the left side.^23^

## Methods

### Trial Design and Participants

This open-label, randomized, multicenter, parallel-group, superiority trial was conducted at 10 FETO centers and 26 neonatal care centers in Belgium, the United Kingdom, France, Spain, Canada, Italy, Japan, the United States, Germany, the Netherlands, Switzerland, and Poland (see Table S1 in the Supplementary Appendix, available with the full text of this article at NEJM.org). To participate, FETO centers were required to have performed a minimum of 36 fetoscopies per year (irrespective of indication), to have performed a minimum of 15 FETO procedures at the time the first participant was recruited, and to have experience with standardized assessment of fetuses with congenital diaphragmatic hernia.^24^

All the women were assessed for eligibility at the FETO centers. The inclusion criteria were a maternal age of 18 years or more, singleton pregnancy, gestational age at randomization of less than 29 weeks 6 days, congenital diaphragmatic hernia on the left side with no other major structural or chromosomal defects, and severe pulmonary hypoplasia, defined as a quotient of the observed-to-expected lung-to-head ratios of less than 25.0%, irrespective of liver position.^8,24^ The exclusion criteria were maternal conditions that would make fetal surgery risky, technical limitations precluding fetal surgery (including those caused by severe maternal obesity or uterine fibroids), an elevated risk of preterm birth (cervical length <15 mm, müllerian anomalies, or placenta previa), and psychological, socioeconomic, or other factors that might prevent adherence to the protocol, which is available at NEJM.org. We kept a log of eligible nonparticipants and their outcomes.

Eligible women received multidisciplinary counseling and standard information on congenital diaphragmatic hernia and FETO,^25^ as well as information about the concept of a randomized trial. Fetoscopic placement of a tracheal balloon was carried out at 27 weeks 0 days to 29 weeks 6 days of gestation. Reversal of occlusion, either by fetoscopy or by ultrasound-guided puncture of the balloon, was scheduled at 34 weeks 0 days to 34 weeks 6 days of gestation.^26^ The women who were assigned to FETO agreed to live near the FETO center for the duration of tracheal occlusion. If preterm birth was imminent, emergency balloon retrieval was performed in utero (as described above), at the time of delivery while the umbilical cord still connected the infant to the placenta, or by direct puncture immediately after delivery.^27^ After balloon removal, the women were given the option of either delivering in the FETO center or returning home for delivery in their local tertiary referral hospital. In either case, postnatal care was standardized according to international consensus guidelines and was the same for both groups.^21,22^

Approval for the trial was obtained from the relevant research ethics committees and competent authorities in each country. The statistical analysis plan is available with the protocol. The first author vouches for the fidelity of the trial to the protocol and for the accuracy and completeness of the data.^28^

### Randomization

After assessment for eligibility, women were randomly assigned, in a 1:1 ratio, to one of the two treatment groups, without stratification factors. Randomization was performed by a fetal medicine specialist using a purposely developed secure website. Block randomization was used for an equal distribution per group at every analysis. The randomization sequence was generated by the statistician.

### Outcome Measures

The primary outcome was survival to discharge from the neonatal intensive care unit (NICU). Secondary and exploratory outcomes were operative and pregnancy complications, fetal survival, survival to 6 months of age, and neonatal complications (Table S2).

### Statistical analysis

We used a group-sequential design and five interim analyses to allow for early stopping for superiority, with a two-sided alpha level of 5% with O’Brien–Fleming stopping rules and a power of 80%.^29^ On the basis of previous studies, the sample-size calculation assumed that survival to discharge from the NICU would be 50% in the FETO group and 25% in the expectant care group.^8,20^ A total of 116 participants (58 in each group) would be required if the trial was not stopped early. No formal boundaries for futility were considered.

We analyzed the primary outcome using the z test for unpaired proportions according to the intention-to-treat principle. A secondary analysis was performed according to the per-protocol principle. No formal significance testing was performed in the analyses of the secondary and exploratory outcomes. We report relative risks, differences in percentages, and differences in medians with 95% confidence intervals because there was no adjustment for multiplicity in the analyses of secondary and exploratory outcomes. These confidence intervals should not be used to infer definitive treatment effects. Because the trial was stopped early, we later performed post hoc analyses that included participants who had undergone randomization but for whom outcome data were not yet available at the time of the third interim analysis; these participants were referred to as “overrunning participants.”^30^ The statistical analysis plan and additional details regarding sample-size considerations for the primary outcome are provided in the protocol.

## Results

### Trial Participants

**Figure 1:**
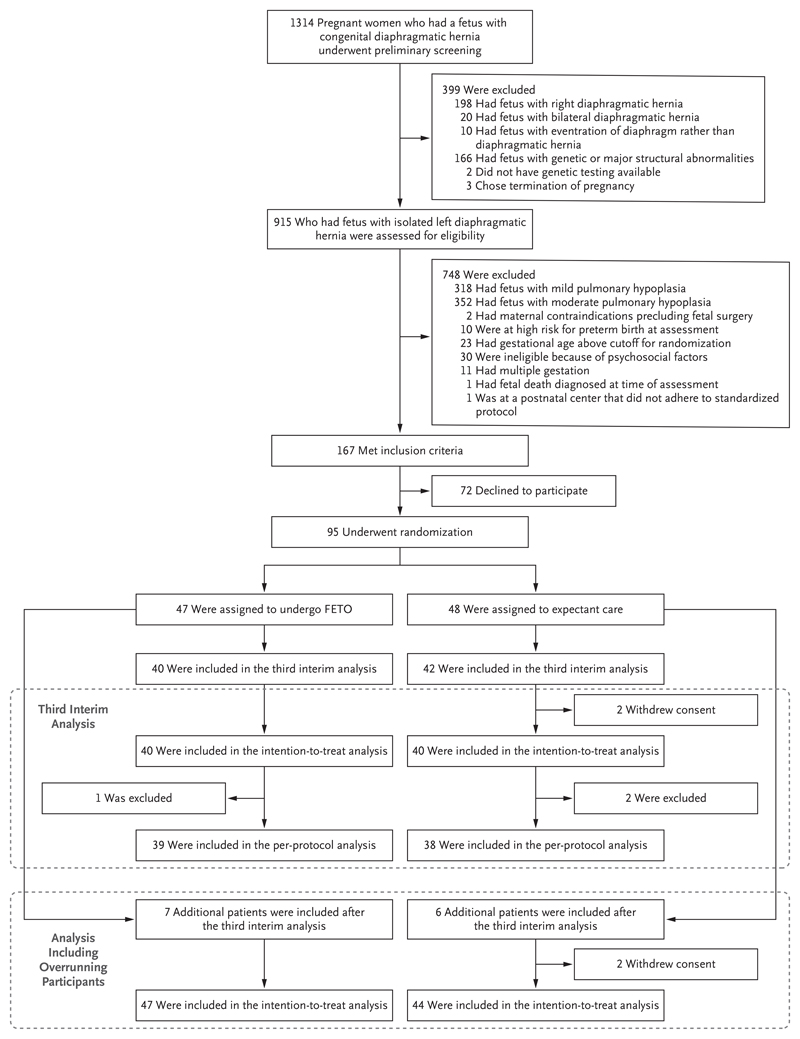
Screening, Randomization, and Analysis. One participant in the fetoscopic endoluminal tracheal occlusion (FETO) group was excluded from the perprotocol analysis because after the FETO procedure was performed, a diagnosis of tetrasomy 12p was made in the fetus, after which the parents opted for palliative care for the infant after birth. Two participants in the expectant care group who requested termination of pregnancy were excluded from the per-protocol analysis. Overrunning participants were those who had undergone randomization after recruitment of the 80 patients required for the third interim analysis and before the results of that analysis became available and recruitment was concluded.

Starting in February 2011, a total of 1314 women carrying fetuses with congenital diaphragmatic hernia underwent preliminary assessment, and 167 met the inclusion criteria; of these women, 95 (57%) provided written informed consent to participate and were assigned to FETO (47 women) or expectant care (48 women) (Fig. 1). Four participants subsequently withdrew consent for participation and data collection. On March 3, 2020, the data and safety monitoring committee stopped the trial for efficacy at the third interim analysis, and enrollment was stopped. The results in 80 participants (40 in each group) are the primary focus of the current article. There were no obvious differences between the FETO group and the expectant care group with respect to baseline characteristics (Table 1).

**Figure 2:**
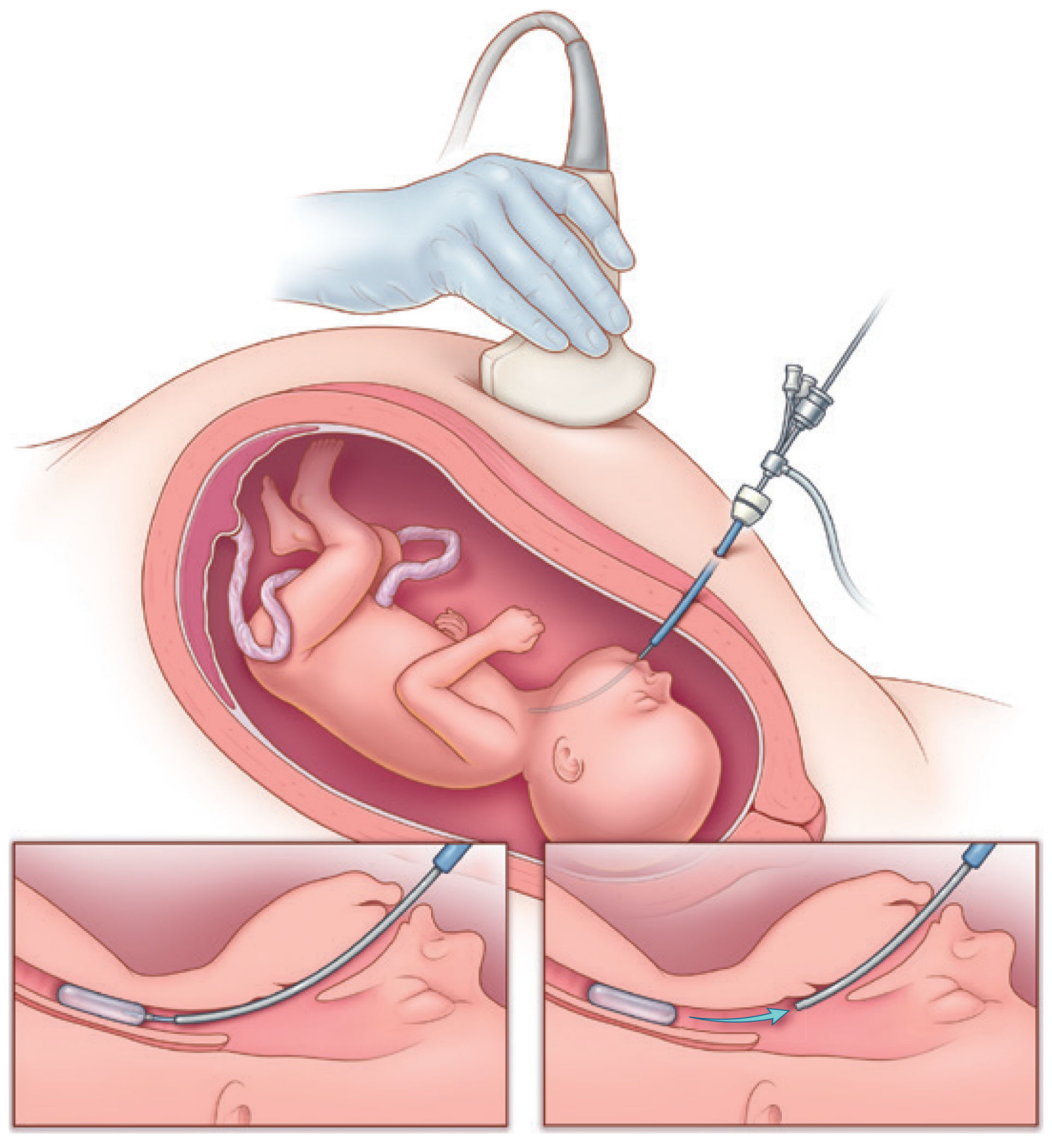
FETO Procedure. After the administration of local anesthesia to the mother and administration of medication to the fetus, the fetoscope is inserted into the amniotic cavity, into the fetal mouth, and eventually into the fetal trachea. In the fetal trachea, the catheter, which has been loaded with a balloon, is advanced to position the balloon between the vocal cords and the carina (bottom left). Once inflated, the balloon is detached, and the fetoscope is withdrawn (bottom right). Adapted from a drawing by Myrthe Boymans, University Hospitals KU Leuven, Leuven, Belgium.

In the FETO group, the balloon was successfully inserted into the trachea in all fetuses (Fig. 2). In most cases, the procedure was carried out while the women were under local or regional anesthesia, but two women received general anesthesia because of anxiety (Table S3).

No maternal complications occurred during FETO. There were five spontaneous balloon deflations. In 1 participant, a new balloon was inserted at the time deflation was identified (at 29 weeks 3 days of gestation), but this balloon spontaneously deflated 1 week later. In the other participants, balloon deflation was detected just before removal (at 33 weeks 1 day) or during removal (at 30 weeks 2 days, 34 weeks 0 days, and 34 weeks 2 days). In 4 participants, removal was not attempted because the parents opted for withdrawal of care (in 2 participants) or because balloon deflation was detected before planned removal (in 2 participants). The balloon was removed as originally planned during the 34th week of gestation in 22 of 38 participants (58%); but in 14 participants (37%), balloon removal was performed earlier than planned, mainly because the mothers had spontaneous onset of labor or preterm, prelabor rupture of membranes. The median interval between balloon insertion and removal was 34 days (interquartile range, 28 to 39), and the median change in the quotient of the observed-to-expected lung-to-head ratios from baseline was 67.0% (interquartile range, 24 to 121). In 38% of participants, delivery occurred within 24 hours after balloon removal. The preferred method for balloon removal was fetoscopy, but in 10 of 38 participants (26%), alternative methods were used (Table S3).

### Primary Outcome

A total of 16 of 40 infants (40%) in the FETO group and 6 of 40 infants (15%) in the expectant care group survived to discharge from the NICU (relative risk, 2.67; 95% confidence interval [CI], 1.22 to 6.11; P = 0.009) (Table 2). The per-protocol analysis yielded similar results: 16 of 39 infants (41%) in the FETO group and 6 of 38 infants (16%) in the expectant care group survived to discharge from the NICU (relative risk, 2.60; 95% CI, 1.19 to 5.93). In the intention-to-treat analysis, one neonatal death occurred after emergency delivery because of fetoscopic placental laceration from balloon removal, and one neonatal death occurred due to problems associated with balloon removal.

### Secondary Outcomes

Survival to 6 months of age was identical to the survival to discharge from the NICU (relative risk, 2.67; 95% CI, 1.22 to 6.11). Preterm, prelabor rupture of membranes occurred in 19 of 40 women (47%) in the FETO group and in 4 of 38 (11%) in the expectant care group (relative risk, 4.51; 95% CI, 1.83 to 11.9). Preterm birth occurred in 30 of 40 women (75%) in the FETO group and in 11 of 38 women (29%) in the expectant care group (relative risk, 2.59; 95% CI, 1.59 to 4.52). The median gestational age at delivery was 34 weeks 4 days and 38 weeks 3 days in the two groups, respectively, and the median birth weight in the FETO group was 481 g lower than that in the expectant care group (Table 3). There were no obvious between-group differences in the incidence of adverse neonatal outcomes. Results for outcomes in infants who survived to discharge from the NICU are reported in Table S4; these results are presented descriptively because they are not representative of the assigned intervention and are subject to bias. Table S5 shows details regarding the chromosomal or structural defects that were not recognized at randomization (in 2 infants in the FETO group and in 1 in the expectant care group).

### Adverse Events

Table 3 shows the adverse events that occurred in the safety population, which included all the participants who underwent randomization and received the prenatal intervention to which they were assigned. Aside from preterm, prelabor rupture of membranes and preterm delivery, there was one case of placental abruption in each group: one case occurred 22 days (30 weeks 5 days) after FETO and 3 days after membrane rupture, and one case occurred at 35 weeks 3 days in the expectant care group.

In the FETO group, there was one case of procedure-related placental laceration from fetoscopic balloon removal, leading to hemorrhage, fetal bradycardia, delivery by emergency cesarean section, and neonatal death during resuscitation. One participant, who moved away from the FETO center, presented to her local unit at 33 weeks 6 days of gestation in preterm labor and with intact membranes; postnatal puncture was unsuccessful and resulted in neonatal death. Tracheomalacia was diagnosed at 10 months of age in one infant; this child, who was still dependent on oxygen at 3 years of age, had a complex postnatal course, including assisted ventilation for 240 days and two cardiac operations for a ventricular septal defect that was not detected before birth.

### Additional Results

The baseline characteristics of the mothers and infants and the results of post hoc analyses (including the “overrunning” population) are provided in Tables S6 through S10. Among all those infants, the survival to discharge was 36% in the FETO group and 14% in the expectant care group (relative risk, 2.65; 95% CI, 1.21 to 6.09; P = 0.01). Results for 71 eligible participants who did not undergo randomization are shown in Figure S1.

## Discussion

In this multicenter, randomized trial involving women carrying singleton fetuses with severe pulmonary hypoplasia due to isolated congenital diaphragmatic hernia on the left side, prenatal intervention with FETO at 27 to 29 weeks of gestation resulted in significantly higher survival to discharge from the NICU than expectant care (40% vs. 15%). The higher survival in the FETO group was sustained at 6 months of age. However, the risk of preterm, prelabor rupture of membranes was 4.5 times as high in the FETO group as in the expectant care group, and the risk of preterm birth was 2.6 times as high as in the FETO group. No other serious complications occurred in the women, and there were no obvious between-group differences in the duration of stay in the NICU, the duration of ventilatory support, or the incidence of complications related to preterm birth. However, the trial was not powered for these secondary outcomes.

Previous studies have suggested that FETO improves survival among infants with congenital diaphragmatic hernia. Two small, single-center, randomized trials compared fetal tracheal occlusion with expectant care during pregnancy. The first trial included extraluminal or endoluminal tracheal occlusion in 11 fetuses after the mother had undergone laparotomy under general anesthesia and 13 women who received expectant care.^31^ Infants in the tracheal occlusion group were delivered significantly earlier than those with mothers who had received expectant care, but survival was similar in both groups; however, many of the fetuses had not met the current criteria for severe congenital diaphragmatic hernia, as used in the current trial.^8,32^ The second trial involved FETO at 26 to 30 weeks of gestation in 19 fetuses and 19 women who received expectant care^33^; severe hypoplasia was required for eligibility. Infant survival was substantially higher in the FETO group than in the expectant care group (52.6% vs. 5.6%). However, there are some concerns regarding the methods used in the trial, such as the inclusion of fetuses with congenital diaphragmatic hernia on the right side and the fact that severity assessment was not corrected for gestational age.^34^ The magnitude of the increase in survival in the FETO group in the current trial is consistent with previous observational data.^11,20,35–37^ The survival of 15% in the expectant care group was similar to the anticipated survival of 18% on the basis of the quotient of the observed-to-expected lung-to-head ratios at randomization.^8^ Among infants in the expectant care group whose mothers did not undergo randomization, survival was 31% (Fig. S1), but the quotient of the observed-to-expected lung-to-head ratios among these infants was indicative of less severe disease than that among infants whose mothers underwent randomization. In addition, data on pregnancy terminations, preterm births, and fetal abnormalities diagnosed after birth were not available for mothers who did not undergo randomization. Similar considerations apply to other multicenter studies^38^ — including one that used the same postnatal care protocol as that used in the current trial^22,39^ — that showed slightly higher survival in infants with severe congenital diaphragmatic hernia who received expectant care (21 to 25%) than that in the expectant care group in the current trial.

FETO is minimally invasive and has not appeared to have had adverse effects on long-term reproductive outcomes^40^; however, the procedure may not be successful, and it could potentially cause maternal and fetal complications.^41^ The most feared adverse event is an inability to remove the balloon, leading to rapid neonatal death. We previously reported that this is more likely to occur if balloon removal becomes an emergency, rather than being performed as an elective procedure.^20^ Balloons were removed in nine fetuses at a non-FETO center by an inexperienced team, and the removal was problematic in three of these fetuses.^27^ In the current trial, we requested that the participants live near the FETO center during the occlusion period. One mother had moved away, and delivery at a non-FETO center was complicated by failed balloon removal and resulted in neonatal death. We identified five spontaneous balloon deflations; these deflations could potentially compromise any intended therapeutic effect. We and others have previously reported spontaneous balloon deflations, despite testing of both the balloon and the valve before insertion.^20,27^ Our use of the balloon is “off-label”; the indicated use is in an endovascular occlusion system, in which late deflation does not have clinically significant consequences.

Other than preterm, prelabor rupture of membranes (in 47% of participants) and premature birth (in 75%), we did not observe maternal complications with FETO, a finding consistent with our earlier experience.^20^ In a systematic review reporting on 634 FETO procedures, the next most common and potentially severe complication was chorioamnionitis, which was reported in 1.1% of the women.^41^

Limitations of our trial should be noted. A long time period was required to complete the trial, during which the protocols for postnatal care of congenital diaphragmatic hernia may have changed; however, this would not differentially have affected outcomes between the two treatment groups. In addition, management teams were aware of group assignments, but this is unlikely to have affected outcomes. We have information only on short-term outcomes; further study is needed to assess longer-term outcomes after FETO as compared with expectant care for severe congenital diaphragmatic hernia. Although we did not find an increased incidence of adverse outcomes associated with preterm birth in the FETO group, our trial was not powered for these outcomes or for uncommon fetal or maternal complications. Because the trial involved experienced fetal surgery units, the findings should not be generalized to centers without extensive experience in fetoscopy and FETO or to centers that cannot ensure availability of a team that can perform safe and effective balloon retrieval.

This trial involving fetuses with isolated severe congenital diaphragmatic hernia on the left side showed that FETO resulted in increased survival to hospital discharge (and this increase was sustained in the cohort of infants until 6 months of age) but an increased risk of preterm, prelabor rupture of membranes and preterm birth.

## Supplementary Material

Appendix

## Figures and Tables

**Table 1:** Baseline Characteristics of the Mothers and Infants in the Intention-to-Treat Population.*

Characteristic	FETO Group (N = 40)	Expectant Care Group (N = 40)
Median maternal age (IQR) — yr	32.4 (27.6–36.0)	29.9 (25.9–33.8)
Median gestational age at randomization (IQR) — wk	27.7 (26.4–28.6)	27.0 (26.4–28.0)
Nulliparous women — no. (%)	20 (50)	18 (45)
Median BMI (IQR)†	25.3 (21.7–30.1)	24.8 (21.3–30.5)
Cigarette smoker — no. (%)	0	6 (15)
Alcohol use during pregnancy — no. (%)	0	0
Race — no. (%)‡		
White	32 (80)	33 (82)
Asian	5 (12)	3 (8)
Black	1 (2)	4 (10)
Other	2 (5)	0
Findings on ultrasonography at randomization		
Median quotient of observed-to-expected lung-to-head ratios (IQR) —%§	21.0 (19.6–23.3)	21.0 (18.0–23.0)
Intrathoracic liver herniation — no. (%)	36 (90)	35 (88)
Median deepest vertical pocket of amniotic fluid (IQR) — cm	6.6 (5.6–8.0)	6.2 (5.8–7.4)
Median cervical length (IQR) — mm	34 (30–39)	37 (32–39)
Placental position — no. (%)		
Anterior	28 (70)	23 (58)
Posterior	11 (28)	16 (40)
Fundal	1 (2)	1 (2)

* Shown are data up to the time of the third interim analysis, when the trial was stopped. FETO denotes fetoscopic endoluminal tracheal occlusion, and IQR interquartile range.

† The body-mass index (BMI) is the weight in kilograms divided by the square of the height in meters.

‡ Race was reported by the participants.

§ The quotient of observed-to-expected lung-to-head ratios is calculated as the ratio of the ultrasonographic measurement of the observed lung area to head circumference (measured on ultrasonography) divided by the ratio of that which would be expected in a healthy fetus of the same gestational age.

**Table 2:** Outcomes According to Trial Group in the Intention-to-Treat Population.*

Outcome	FETO Group (N = 40)	Expectant Care Group (N = 40)	Relative Risk (95% Cl)	Difference (95% Cl)†
**Primary outcome**				
Survival to discharge from NICU — no. (%)	16 (40)	6 (15)	2.67 (1.22 to 6.11)	25 (6 to 46)
**Secondary and exploratory outcomes**				
Postnatal survival — no. (%)				
To 28 days‡	16 (40)	9 (22)	1.78 (0.92 to 3.56)	18 (–2 to 40)
To 56 days‡	16 (40)	6 (15)	2.67 (1.22 to 6.11)	25 (6 to 46)
To 6 mo	16 (40)	6 (15)	2.67 (1.22 to 6.11)	25 (6 to 46)
To 6 mo without oxygen supplementation	9 (22)	3 (8)	3.00 (0.96 to 9.76)	15 (–2 to 33)
Preterm, prelabor rupture of membranes‡§				
Median gestational age (1QR) — wk	32.0 (30.4 to 33.9)	35.9 (34.6 to 36.2)		–3.9 (–5.1 to 0.6)
Rupture of membranes at <37 wk — no./total no. (%)	19/40 (48)	4/38 (11)	4.51 (1.83 to 11.9)	37 (19 to 59)
Rupture of membranes at <34 wk — no./total no. (%)	14/40 (35)	1/38 (3)	13.3 (2.46 to 77.5)	32 (15 to 51)
Gestational age at birth‡§				
Median gestational age (IQR) — wk	34.6 (32.2 to 36.6)	38.4 (36.5 to 39.1)		–3.8 (–4.8 to –2.1)
<37 wk — no./total no. (%)	30/40 (75)	11/38 (29)	2.59 (1.59 to 4.52)	46 (29 to 70)
<34 wk — no./total no. (%)	16/40 (40)	0/38		40 (23 to 59)
<32 wk — no./total no. (%)	10/40 (25)	0/38		25 (8 to 41)
Placental abruption — no./total no. (%)	1/40 (2)	1/38 (3)	0.95 (0.10 to 8.92)	0 (–13 to 13)
Neonatal outcomes in live births¶				
Median birth weight (IQR) — g‡‖	2300 (1800 to 2600)	2768 (2486 to 3134)		–481 (–823 to –232)
Neonatal repair of defect — no./total no. (%)	20/38 (53)	14/38 (37)	1.43 (0.86 to 2.42)	16 (–6 to 40)
Use of prosthetic patch for repair — no./total no. (%)	18/20 (90)	11/14 (79)	1.15 (0.84 to 1.74)	11 (–19 to 39)
Median time to repair of defect (IQR) — days	2 (2 to 5)	7 (4 to 9)		–5 (–7 to –1)
ECMO — no./total no. (%)	2/38 (5)	11/38 (29)	0.18 (0.05 to 0.66)	–24 (–43 to –6)

* Shown are data in the intention-to-treat population (unless otherwise specified) up to the third interim analysis, when the trial was stopped. ECMO denotes extracorporeal membrane oxygenation, and NICU neonatal intensive care unit.

† Differences were calculated as the absolute difference in percentages (expressed in percentage points) for dichotomous data or as the difference in medians for continuous data.

‡ This was an exploratory outcome.

§ Two terminations of pregnancy in the expectant care group were excluded.

¶ Two cases of neonatal palliative care in the FETO group and two terminations of pregnancy in the expectant care group were excluded.

‖ One value was missing in the FETO group. For the calculation of the difference in medians, missing values were addressed according to the protocol.

**Table 3:** Adverse Events in the Safety Population.*

Event	FETO Group (N = 40)	Expectant Care Group (N = 38)†
	*number/total number (%)*	
**Serious adverse events**		
Fetal death		
<24 hr after FETO	0/40	NA
Any time during pregnancy	0/40	0/38
Placental abruption		
<24 hr after FETO	0/40	NA
Any time during pregnancy	1/40 (2)	1/38 (3)
Placental laceration from balloon removal‡	1/40 (2)	NA
Neonatal death due to failure of balloon removal§	1/40 (2)	NA
Tracheomalacia¶	1/40 (2)	0/38
Chorioamnionitis‖	0/40	1/38 (3)
Abnormal cardiotocographic findings before labor‖	2/40 (5)	1/38 (3)
Hospital admission due to decreased fetal movements‖	1/40 (2)	1/38 (3)
Hospital admission due to preterm contractions, but delivery at term‖	1/40 (2)	0/38
Preterm, prelabor rupture of membranes <37 wk	19/40 (48)	4/38 (11)
Delivery <37 wk	30/40 (75)	11/38 (29)
Complications related to extraction of the head during breech delivery‖	1/40 (2)	0/38
Death		
Neonatal <28 days	24/40 (60)	29/38 (76)
Between 28 days and 6 months	0/40	3/38 (8)
Perinatal asphyxia, umbilical pH <7.00	1/24 (4)	2/29 (7)
ECMO	2/40 (5)	11/38 (29)
Conditions in infants who survived to discharge		
Bronchopulmonary dysplasia	12/16 (75)	5/6 (83)
Pulmonary hypertension	15/16 (94)	6/6 (100)
Periventricular leukomalacia	1/16 (6)	0/6
Sepsis	10/16 (62)	6/6 (100)
Intraventricular hemorrhage	0/16	0/6
Retinopathy of prematurity	0/16	0/6
Necrotizing enterocolitis	0/12	0/6
**Other adverse events**		
Bleeding resulting from trocar insertion during fetoscopy‖	1/40 (2)	NA
Polyhydramnios first manifesting at follow-up ultrasonographic examination	12/35 (34)	NE
Pregnancy-induced hypertension‖	0/40	1/38 (3)
Chorioamniotic membrane separation	8/37 (22)	NE
Gastroesophageal reflux in infants who survived to discharge	11/14 (79)	3/6 (50)

* Shown are data up to the third interim analysis, when the trial was stopped. The safety population included all participants who underwent randomization and received the prenatal treatment to which they were assigned. Unless otherwise specified, events were calculated in the full safety population. NA denotes not applicable, and NE not evaluated.

† Two terminations of pregnancy were excluded.

‡ One instance of massive placental bleeding during trocar insertion resulted in emergency cesarean section and neonatal death.

§ One woman had moved away from the FETO center, and the balloon could not be removed at her local unit.

¶ Tracheomalacia was diagnosed in one infant at 10 months of age, but this infant had previously undergone multiple surgeries.

‖ The occurrence or absence of anticipated adverse events was indicated in check boxes (yes or no). Any other event that was not anticipated could be reported in a free-text field. For the events indicated in check boxes, the denominator takes into account missing values.
