# Supplementary material for: Randomized Trial of Fetal Surgery for Severe Left Diaphragmatic Hernia
Source: N Engl J Med. Author manuscript; Available in PMC 2022 Aug 28. (PMC7613453; doi:10.1056/NEJMoa2027030)
Supplement: Appendix [file EMS152844-supplement-Appendix.pdf]

Supplementary Appendix

This appendix has been provided by the authors to give readers additional information about their work.

Supplement to: Deprest JA, Nicolaides KH, Benachi A, et al. Randomized trial of fetal surgery for severe left diaphragmatic hernia. N Engl J Med 2021;385:107-18. DOI: 10.1056/NEJMoa2027030

TOTAL trial for severe hypoplasia

Supplementary appendix

Table of contents of the appendix		
List of investigators		2
Supplementary Figure S1	Outcomes of eligible non-participants	5
Supplementary Table S1	Alphabetical list of FETO and neonatal management centers	6
Supplementary Table S2	List of reported outcomes and safety endpoints	7
Supplementary Table S3	Operative outcomes in patients in the FETO arm at third interim analysis	10
Supplementary Table S4	Secondary neonatal outcomes in survivors to discharge at third interim analysis	11
Supplementary Table S5	Associated anomalies diagnosed after randomization	12
Supplementary Table S6	Baseline Characteristics of the Trial Participants *including overrunning patients*	13
Supplementary Table S7	Outcome according to trial group in the analysis *including overrunning patients*	14
Supplementary Table S8	Occurrence of adverse events in the safety population *including overrunning patients*	16
Supplementary Table S9	Operative outcomes in patients in the FETO arm *including overrunning patients*	18
Supplementary Table S10	Secondary neonatal outcomes in survivors to discharge *including overrunning patients*	19
Supplementary Table S11	Interim analyses	20
Supplementary Table S12	Case load and historical outcomes for postnatal management centers	21

List of investigators

Principal Investigators

Jan A Deprest[1] M.D. Ph.D, Kypros H Nicolaides[2] M.D., Alexandra Benachi[3] M.D. Ph.D., Eduard Gratacos[4] M.D. Ph.D., Greg Ryan[5] M.D., Nicola Persico[6] M.D. Ph.D., Haruhiko Sago[7] M.D. Ph.D., Anthony Johnson[8] M.D., Miroslaw Wielgos[9] M.D. Ph.D., Christoph Berg[10] M.D. Ph.D., Ben Van Calster[1] Ph.D., Francesca M Russo[1] M.D. Ph.D.

Data Monitoring and Safety Committee and data-managers

Paul Lewi †[1] , Ph.D., Tim Van Mieghem [1,5] M.D. Ph.D., Philip LJ DeKoninck [1,11] M.D. Ph.D., Hugo Devlieger[1] M.D. Ph.D., Michael Harrison[12] , M.D. Ph.D., Francois I Luks[13] M.D. Ph.D., Angélique Rezer[1] LL.M., Kris Dierickx[1] Ph.D., Beverley Power[14]

External committee drafting neonatal guidelines

Dick Tibboel[11] M.D. Ph.D., Thomas Schaible[15] M.D. Ph.D., Laurent Storme[16] M.D. Ph.D.

Affiliations

[1] The University Hospitals KU Leuven, Leuven, Belgium; [2] King's College Hospital, London, UK; [3] Hospital Antoine Béclère, Université Paris Saclay Clamart, France; [4] Hospital Clinic and Sant Joan de Deu, Barcelona, Spain; [5] Mount Sinai Hospital, Toronto, Canada; [6] Hospital Maggiore Policlinico, Milano, Italy; [7] National Center for Child Health and Development, Tokyo, Japan; [8] Children's Hermann Memorial Hospital, Houston, TX, USA; [9] Medical University of Warsaw, Poland; [10] University Hospital Bonn, Bonn, Germany; [11] Erasmus Medical Centre Rotterdam, Rotterdam, The Netherlands; [12] University of California at San Francisco, CA, USA; [13] Hasbro Children's Hospital, Providence, RI, USA; [14] C.D.H. UK Parent Organization, London, UK; [15] Mannheim University Hospital, Mannheim, Germany; [16] University Medical Centre Lille, France;

† Prof P. Lewi deceased during the trial

Other TOTAL trial for severe CDH collaborators

The University Hospitals KU Leuven, Leuven, Belgium - Roland Devlieger M.D Ph.D., Karel Allegaert, M.D. Ph.D., Herbert Decaluwé M.D. Ph.D.; **King's College Hospital, London, UK** - Ramona Cazacu M.D., Theodore Dassios M.D., Shailesh Patel M.D.; **Hôpital Bicêtre. AP-HP. Université Paris Saclay, France** – Anne-Gael Cordier M.D. Ph.D., Mostafa Mokhtari M.D., Jérome Nevoux M.D.; **Hospital Clinic and Hospital Sant Joan de Deu, Universitat de Barcelona, IDIBAPS and CIBERER, Barcelona, Spain** – Joseph Maria Martinez M.D., Olga Gomez M.D., Jordi Prat M.D.; **Mount Sinai Hospital, Toronto, ON, Canada** – Nimrah Abbasi M.D., Karel O'Brien M.D.; **Hospital for Sick Children, Toronto, ON, Canada** – Priscilla P.L.

Chiu, M.D. Ph.D.; **Ospedale Maggiore Policlinico, Milan, Italy** – Isabella Fabietti M.D., Fabio Mosca M.D., Ernesto Leva M.D.; **National Center for Child Health and Development, Tokyo, Japan** – Seiji Wada M.D. Ph.D., Yushi Ito M.D.; **Osaka University Graduate School of Medicine, Osaka, Japan** – Masayuki Endo M.D. Ph.D.; **University of Texas Health Science Center/ Children's Memorial Hermann, Houston, TX, USA** – Mary Austin M.D., Suzanne M. Lopez M.D., Kuojen Tsao M.D.; **Medical University of Warsaw, Warsaw, Poland** – Przemyslaw Kosinski M.D., Bozena Kociszewska-Najman M.D., Andrzej Kaminski M.D.; **Universitätsklinikum Bonn, Bonn, Germany** – Brigitte Strizek M.D., Andreas Müller M.D., Andreas Heydweiller M.D.; **CHRU Lille, Lille, France** – Veronique Houfflin-Debarge M.D. Ph.D., Dyuti Sharma M.D. Ph.D.; **Erasmus MC University Medical Center, Rotterdam, The Netherlands** – Alex J. Eggink M.D. Ph.D., Rene M.H. Wijnen M.D. Ph.D.; **Strasbourg University Hospital, Strasbourg, France** – Nicolas Sananes M.D. Ph.D., Pierre Kuhn M.D., François Becmeur M.D.; **St George's University Hospitals NHS Foundation Trust, London, UK** – Basky Thilaganathan M.D. Ph.D., Nigel Kennea M.D. Ph.D., Bruce Okoye M.D.; **Radboudumc Amalia Children's Hospital, Nijmegen, The Netherlands** – Mallory Woiski M.D., Willem P. de Boode M.D., Horst Daniels-Scharbatke M.D.; **Bambino Gesù Children's Hospital, Rome, Italy** – Anita Romiti M.D., Irma Capolupo M.D., Laura Valfre M.D.; **Saint Luc UCL, Brussels, Belgium** – Jean-Marc Biard M.D. Ph.D, Catheline Hocq M.D., Catherine de Magnée M.D.; **University Hospital Zurich, Zurich, Suisse** – Nicole Ochsenbein-Kölble M.D. Ph.D., Ueli Möhrlen M.D. Ph.D.; **University Hospital Center Montpellier, Montpellier, France** – Florent Fuchs M.D. Ph.D., Odile Pidoux M.D., Dominique Forgues M.D.; **CHU Sainte Justine Université de Montréal, Montréal, Canada** – Lucie Morin M.D., Anne-Monique Nuyt M.D., Dickens St Vil M.D.; **Hospices civils de Lyon, Lyon, France** – Jerome Massardier M.D., Sebastien Blanc M.D., Frederic Hameury M.D.; **CHU Clermont-Ferrand, Clermont-Ferrand, France** – Amelie Delabaere M.D., Karen Coste M.D., Maguelonne Pons M.D.; **CHU Toulouse, Toulouse, France** – Marion Groussolles M.D., Marie-Odile Marcoux M.D., Olivier Abbo M.D.; **Hôpital Trousseau, Paris, France** – Jean-Marie Jouannic M.D. Ph.D., Julia Guilbert M.D., Sabine Irtan M.D. Ph.D.; **Hôpital Robert Debré, Paris, France** – Jonathan Rosenblatt M.D., Michael Levy M.D., Elisabeth Carricaburu M.D.; **Medway Maritime Hospital, Gillingham, UK** – Ranjit Akolekar M.D.; **Luton and Dunstable Hospital, Luton, UK –** Jacqueline Bamfo M.D.; **Addenbrooke's Hospital, Cambridge, UK** – Jennifer Brewster M.D.; **William Harcey Hospital, Ashford, UK** – Sarah Chissel M.D.; **Homerton University Hospital, London, UK** – Simona Cicero M.D.; **Leeds Teaching Hospitals NHS Trust, Leeds, UK** – Kelly Cohen M.D.; **Universitary Hospital Bristol, Bristol, UK** – Mark Denbow M.D.; **Royal London Hospital, London, UK** – Elena Greco M.D.; **The John Radcliffe Hospital, Oxford, UK –** Lawrence Impey M.D.; **Nagoya University Graduate School of Medicine, Nagoya, Japan** – Tomomi Kotani M.D.; **University College London Hospitals NHS**

Foundation, London, UK – Pranav Pandya M.D.; **Eastbourne District General Hospital, Eastbourne, UK** – Nicky Roberts M.D.; **Southend University Hospital NHS Foundation Trust, Southend-on-Sea, UK** – Mandeep Singh M.D.; **Royal Victoria Infirmary, Newcastle upon Tyne, UK** – Stephen Sturgiss M.D.; **Lewisham University Hospital, London, UK** – Devi Subramanian M.D.; **Osaka Women's and Children's Hospital, Osaka, Japan** – Noriaki Usui M.D. Ph.D.; **Leiden University Medical Center, Leiden, The Netherlands** – Inge Van Kamp M.D. Ph.D.; **Maastricht University Medical Centre, Maastricht, The Netherlands** – Christine Willekes M.D. Ph.D.; **Royal Stoke University Hospital, Stoke-on-Trent, UK** – Pensee Wu M.D.;

Click to go to top of document: table of contents

Supplementary Figure S1: Characteristics and outcomes in eligible non-participants to the study

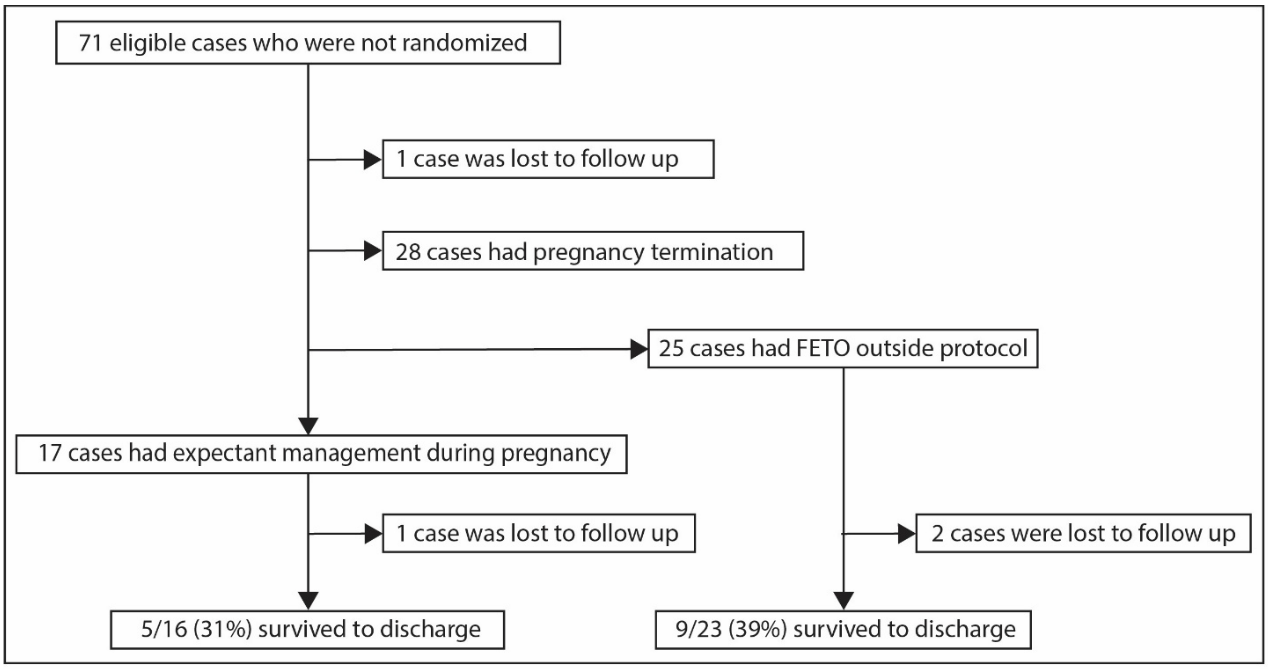

	n	O/E LHR	Liver herniation	Lost to follow up	Survival to discharge from NICU
Expectant management	17	23.0 (19.1-24.0)	12/16* (75%)	1/17 (6%)	5/16 (31%)
FETO	25	21.0 (18.2-23.6)	22/25 (88%)	2/25 (8%)	9/23 (39%)

Continuous variables are reported as medians and interquartile ranges and categorical variables as absolute numbers and percentages. *One missing value.

Abbreviations: FETO: fetoscopic endoluminal tracheal occlusion; O/E LHR: observed to expected lung-to-head ratio; NICU: neonatal intensive care unit.

Click to go to top of document: table of contents

Supplementary Table S1: Alphabetical list of FETO and their collaborating neonatal management centers

FETO centers	Neonatal management centers
Hospital Clinic, University of Barcelona, Barcelona, Spain	Hospital San Joan de Deu, Barcelona, Spain
Universitätsklinikum Bonn, Germany	Universitätsklinikum Bonn, Germany
Hôpital Antoine Béclerè, Clamart, France	Hôpital Bicêtre, Paris, France<br>CHU Clermont-Ferrand, France<br>CHRU Lille, France<br>CHRU Montpellier, Montpellier, France<br>HFME Lyon, Lyon, France<br>Hôpitaux de Toulouse Paule de Viguier, Toulouse, France<br>Hôpital Trousseau, Paris, France<br>Hôpital Robert Debré, Paris, France
University of Texas Health Science Center / Children's Memorial Hermann, Houston, TX, USA	University of Texas Health Science Center / Children's Memorial Hermann, Houston, TX, USA
University Hospitals Leuven, Belgium	CHRU Lille, France<br>Erasmus MC, Rotterdam, The Netherlands<br>Ospedale Pediatrico Bambino Gesù, Rome, Italy<br>Radboudumc/Amalia Children's Hospital Nijmegen, The Netherlands<br>Schiltigheim, Centre Medico-Chirurgical et Obstetrical, Strasbourg, France<br>UCL St Luc, Brussels , Belgium<br>Universitätsklinik für Frauenheilkunde Wien, Wien, Austria<br>University Hospital Leipzig, Leipzig, Germany<br>University Hospitals Leuven, Leuven, Belgium<br>University Hospital Zurich, Switzerland
King's College Hospital, London, UK	Addenbrooke's Cambridge, UK<br>John Radcliffe Hospital, Oxford, UK<br>King's College Hospital, London, UK<br>Leeds Teaching Hospitals NHS Trust, Leeds, UK<br>Liverpool Women's Hospital, Liverpool, UK<br>Royal London Hospital, UK<br>Royal Sussex County, Brighton, UK<br>Royal Stoke University Hospital, Stoke-on-Trent, UK<br>St.George's Hospital, London, UK<br>University College Hospital, London, UK<br>Universitary Hospital Bristol, Bristol, UK
Ospedale Maggiore Policlinico, Milano, Italy	Ospedale Maggiore Policlinico, Milano, Italy
Mount Sinai Hospital, Toronto, ON, Canada	SickKids Hospital, Toronto, Canada<br>CHU Sainte Justine Université de Montréal
1st Department of Obstetrics and Gynecology, Medical University of Warsaw, Poland	1st Department of Obstetrics and Gynecology, Medical University of Warsaw, Poland
Japan National Center for Child Health and Development, Tokyo, Japan	Japan National Center for Child Health and Development, Tokyo, Japan

Click to go to top of document: table of contents

Supplementary Table S2: List of primary, secondary and exploratory outcome measures, and safety endpoints prospectively collected via the electronic record forms.

**Primary outcome**	**Definition**
Survival to discharge	Alive at discharge from NICU
**Secondary outcomes**	
Change in observed / expected LHR from baseline	(value before balloon removal- baseline value / baseline value)*100 (%)
Change in MRI O/E total lung volume from baseline	(value before balloon removal- baseline value / baseline value)*100 (%)
Bronchopulmonary dysplasia according to Jobe and Bancalari[1]	Need for supplemental oxygen FiO2 >0.21 for at least 28 days. This is assessed at:<br>>28 days but <56 days postnatal age or discharge, whichever comes first; for infants born ≥ 32 weeks<br>36 weeks postmenstrual age or discharge, whichever comes first; for infants born < 32 weeks
Grading of oxygen dependency according to Jobe and Bancalari[1]	Measured<br>- at d56 postnatal age or discharge, whichever comes first, for infants born ≥32w<br>- at 36w postmenstrual age or discharge, whichever comes first, for infants born <32w<br>Categorized as<br>- Mild BPD: FiO2 0.21<br>- Moderate BPD: FiO2 0.22-0.29<br>- Severe BPD: FiO2 >0.30, and/or CPAP and/or mechanical ventilation
Pulmonary hypertension	Based on evidence on cardiac ultrasound of predominant unidirectional right to left shunt.
ECMO	Use of ECMO
Length of stay in NICU	Number of days from birth until discharge from NICU
Days of ventilatory support	Number of days of ventilatory support: mechanical ventilation, CPAP, optiflow. Low flow oxygen was not considered ventilatory support
Periventricular leukomalacia	As diagnosed by postnatal center using local criteria
Neonatal sepsis	As diagnosed by postnatal center using local criteria
Intraventricular hemorrhage > grade III in survivors to discharge	As diagnosed by postnatal center using local criteria
Retinopathy of prematurity	As diagnosed by postnatal center using local criteria
Days until full enteral feeding	Number of days from birth until full enteral feeding was established
Gastro-esophageal reflux	Above 1/3 of the esophagus on clinically indicated radiologic study
Day of postnatal surgery	Number of days from birth until the day of postnatal surgery
Use of patch	Use of patch at postnatal surgery
Defect size according to CDH study group[2]	A: small defect, with muscular edges all around<br>B: <50% chest wall involvement<br>C: > 50% chest wall involvement<br>D: total agenesis of diaphragm
Date of postnatal death	Date
Survival to 6 months of age	Survival at 6 months of age
Supplemental oxygen at 6 months of age	Any supplemental oxygen administration
**Exploratory outcomes**	
Gestational age at FETO and balloon removal	Gestational age in weeks and days

Successful placement of balloon	Balloon positioned between carina and vocal cords
Anesthesia during FETO and balloon removal	Local / neuraxial / general
Chorioamniotic membrane separation	Echolucent space between membranes and uterine wall evidenced on ultrasound examination
Spontaneous deflation of balloon	No balloon visible inside the fetal trachea at follow up ultrasound or at the time of fetoscopic removal
Polyhydramnios after FETO	Presence of a deepest vertical pocket of ≥ 8cm on ultrasound, diagnosed at any time point after FETO procedure
Gestational age at balloon removal	Gestational age in weeks and days
Emergency balloon removal	Balloon removal prior to scheduled date because of threatened preterm birth
Method of balloon removal	Fetoscopy / ultrasound guided puncture / postnatal
Interval balloon removal and delivery <24hrs	Duration between balloon removal and birth is less than 24 hours
Preterm prelabor rupture of membranes (PPROM) < 37 wks	Presence of ruptured membranes prior to 37 weeks gestational age
PPROM < 34 wks	Presence of ruptured membranes prior to 34 weeks gestational age
Gestational age at PROM	Gestational age at membrane rupture, in weeks and days
Placental abruption	Occurrence of placental abruption
Gestational age at birth	Gestational age at birth, in weeks and days
Gestational age at birth < 32+0 wks	Birth < 32+0 wks
Gestational age at birth < 34+0 wks	Birth < 34+0 wks
Gestational age at birth ≥ 37+0 wks	Birth ≥ 37+0 wks
Live born	Alive at birth
Birthweight	Birthweight in grams
Necrotizing enterocolitis	Diagnosed by postnatal center using local criteria
Tracheomalacia	Diagnosed by postnatal center using local criteria
Survival to 28 days	Alive at day 28 after birth
Survival to 56 days	Alive at day 56 after birth
**Exploratory safety endpoints**	
IUFD < 24hrs after FETO	Occurrence of intrauterine fetal death within 24hrs after FETO procedure
Placental abruption < 24hrs after FETO	Occurrence of placental abruption within 24hrs after FETO procedure
Balloon removal issues	Any deviation from the normal course, or problem with balloon removal
IUFD at any point in pregnancy	Occurrence of intrauterine fetal death at any time point during pregnancy
Placental abruption at any point in pregnancy	Occurrence of placental abruption at any time point during pregnancy
Tracheomalacia	Diagnosed by postnatal center using local criteria
PPROM <37 wks	Presence of ruptured membranes prior to 37 weeks gestational age
Delivery <37 wks	Birth < 37+0 wks
Neonatal death <28 days	Neonatal death in the first 27 days
Death between 28 days and 6 months	Death between 28 days and 6 months after birth
Perinatal asphyxia (umbilical pH <7.00)	Umbilical artery pH <7.00
ECMO	Use of ECMO
Bronchopulmonary dysplasia according to Jobe and Bancalari[1] in survivors to discharge	Need for supplemental oxygen FiO2 >0.21 for at least 28 days. This is assessed at:

	- >28 days but <56 days postnatal age or discharge, whichever comes first; for infants born ≥ 32 weeks<br>- 36 weeks postmenstrual age or discharge, whichever comes first, for infants born < 32 weeks
Pulmonary hypertension in survivors to discharge	Based on evidence on cardiac ultrasound of predominant unidirectional right to left shunt.
Periventricular leukomalacia in survivors to discharge	As diagnosed by postnatal center using local criteria
Neonatal sepsis in survivors to discharge	As diagnosed by postnatal center using local criteria
Intraventricular hemorrhage > grade III in survivors to discharge	As diagnosed by postnatal center using local criteria
Retinopathy of prematurity in survivors to discharge	As diagnosed by postnatal center using local criteria
Polyhydramnios after FETO	Presence of a deepest vertical pocket of ≥ 8cm on ultrasound, diagnosed at any time point after FETO procedure
Chorioamniotic membrane separation	Echolucent space between membranes and uterine wall evidenced on ultrasound examination
Any additional adverse event (free text field)	

[1]*Jobe AH, Bancalari E. Bronchopulmonary dysplasia. Am J Respir Crit Care Med. 2001 Jun;163(7):1723-9.*
[2]*Tsao K, Lally KP. The congenital diaphragmatic hernia study group: a voluntary international registry. Semin Pediatr Surg 2008;17:90-7.*

Click to go to top of document: table of contents

Supplementary Table S3: Operative outcomes in patients in the FETO arm at third interim analysis.

Characteristic	
**FETO procedure (n=40)**	
Gestational age at FETO – wk	28.6 (28.0 – 29.0)
Anesthesia: local – no. (%)	30/40 (75)
Anesthesia: neuraxial block – no. (%)	8/40 (20)
Anesthesia: general – no. (%)	2/40 (5)
**Balloon removal procedure (n=38*)**	
Gestational age at balloon removal – wk	34.0 (32.8 – 34.3)
Emergency balloon removal – no. (%)†	14/38 (37)[a]
Gestational age – wk	32.4 (31.2 – 33.3)[a]
Method: fetoscopy – no. (%)	5/14 (36)[a]
Method: ultrasound guided puncture – no. (%)	2/14 (14)
Postnatal – no. (%)	7/14 (50)
Elective balloon removal† – no. (%)	22/38 (58)[b]
Gestational age – wk	34.3 (34.0 – 34.5)[b]
Method: fetoscopy – no. (%)	21/22 (95)[b]
Method: ultrasound guided puncture – no. (%)	1/22 (5)
No removal because of spontaneous deflation - no. (%)	2/38 (5)
**Interval between balloon insertion and removal (n=38*)**	
Duration of tracheal occlusion – days	34 (28 – 39)[c]
Change in O/E LHR from baseline – %	67 (24 – 121)[d]
**Interval between balloon removal and delivery (n=40)**	
<24 hours - no. (%)	15/40 (38)

Continuous variables are reported as medians and interquartile ranges, categorical variables as absolute numbers and percentages. Abbreviations: FETO: fetoscopic endoluminal tracheal occlusion; GA: gestational age; O/E LHR: observed to expected lung-to-head ratio. All exploratory outcomes except for change in O/E LHR from baseline.

*In two cases balloon removal was not attempted at birth, because care was withdrawn. [a]Including one fetus where the balloon was deflated. [b]Including two fetuses where the balloon was deflated. [c]excluding five spontaneous balloon deflations; [d]four (10%) missing values. Abbreviations: FETO: fetoscopic endoluminal tracheal occlusion; † Reasons given were: prelabor membrane rupture (n=9), preterm labor (n=4), fetal distress (n=1).

Click to go to top of document: table of contents

Supplementary Table S4: Secondary neonatal outcomes in survivors to discharge at third interim analysis

Outcome	FETO (n=16)	Expectant (n=6)
Days to neonatal repair of defect	2 (2-5)	4 (1-8)
ECMO days in survivors receiving ECMO (n=3)	nc (n=1)[a]	nc (n=2)[a]
Days of ventilatory support	49 (24-93)	40 (18-53)
Days to full enteral feeding[b]	30 (18-45)	31 (18-46)
Bronchopulmonary dysplasia – no. (%)	12/16 (75)	5/6 (83)
Mild	4/12 (33)	2/5 (40)
Moderate	6/12 (50)	1/5 (20)
Severe	2/12 (17)	2/5 (40)
Pulmonary hypertension – no. (%)	15/16 (94)	6/6 (100)
Periventricular leukomalacia – no. (%)	1/16 (6)	0/6 (0)
Sepsis – no. (%)	10/16 (63)	6/6 (100)
Intraventricular hemorrhage > grade III – no. (%)	0/16 (0)	0/6 (0)
Retinopathy of prematurity > grade 3 – no. (%)	0/16 (0)	0/6 (0)
Necrotizing enterocolitis – no. (%)[c] °	0/12 (0)	0/6 (0)
Gastroesophageal reflux – no. (%)[b]	11/14 (79)	3/6 (50)
NICU days	68 (35-106)	85 (74-107)

Results are reported as absolute numbers and percentages (categorical variables) or as medians and interquartile ranges (continuous variables). °Exploratory outcomes. [a]nc: not calculated because n too low or percentage of 0 in both arms. [b]Two (9%) missing values. [c]Four (18%) missing values.

Click to go to top of document: table of contents

Supplementary Table S5: associated anomalies diagnosed after randomization

Diagnosis	Time of diagnosis	Group	Included in per-protocol analysis (Yes/No)
Tetrasomy 12p	Prenatal	FETO	No
Large cardiac ventricular septum defect	Postnatal	FETO	Yes
Golz Syndrome	Postnatal	Expectant	Yes

Summary of severe structural, genetic or syndromic diagnoses that were made after randomization. Inclusion into the per-protocol analysis was decided by the DMSC in consultation with two geneticists. “Postnatal” (column 2) indicates the diagnosis was made during the postnatal period, though prior to discharge from the NICU.

Click to go to top of document: table of contents

Supplementary Table S6: Baseline characteristics of the trial participants including overrunning patients**.**

**Characteristic**	**FETO (n= 47)**	**Expectant (n= 44)**
Maternal age – years	32.1 (27.3 - 35.3)	29.4 (25.9 – 33.8)
Gestational age at randomization– wk	27.7 (26.6 – 28.6)	27.0 (26.4 – 28.0)
Nulliparous – no. (%)	25 (53)	20 (45)
Body mass index – Kg/m^2	24.1 (21.9 – 29.7)	24.3 (21.7 – 30.5)
Cigarette smoker – no. (%)	0 (0)	6 (14)
Alcohol use during pregnancy – no. (%)	0 (0)	0 (0)
Race or ethnicity group – no. (%)		
White	34 (72)	33 (75)
Asian	6 (13)	4 (9)
Black	2 (4)	4 (9)
Other	2 (4)	0 (0)
Ultrasound findings at randomization		
Observed/expected Lung-to-Head-Ratio - %	21.0 (19.9 – 24.0)	21.0 (18.0 – 23.0)
Intrathoracic liver herniation – no. (%)	42 (89)	38 (86)
Deepest vertical pocket of amniotic fluid– cm	6.5 (5.6 – 8.0)	6.2 (5.8 – 7.4)
Cervical length– mm	34 (30-39)	36 (32-39)
Placenta anterior – n (%)	33 (70)	24 (55)
Placenta posterior – n (%)	13 (28)	19 (43)
Placenta fundal – n (%)	1 (2)	1 (2)

Click to go to top of document: table of contents

Supplementary Table S7: Outcome according to trial group in the analysis including overrunning patients

Outcome	FETO (n=47)	Expectant (n=44)	Relative Risk (95% CI)	Difference (95%CI)[#]
**Primary outcome**				
Survival to discharge from NICU – no. (%)	17/47 (36)	6/44 (14)	2.65 (1.21-6.09)	23 (5 to 42)
**Secondary outcomes**				
**Postnatal survival**				
Survival to 28 days – no. (%)°	17/47 (36)	9/44 (20)	1.77 (0.91-3.56)	16 (-3 to 36)
Survival to 56 days – no (%)°	17/47 (36)	6/44 (14)	2.65 (1.21-6.09)	23 (5 to 42)
Survival to 6 months – no. (%)	17/47 (36)	6/44 (14)	2.65 (1.21-6.09)	23 (5 to 42)
Survival to 6 months without oxygen – no. (%)	10/47 (21)	3/44 (7)	3.12 (1.00-10.1)	14 (-1 to 31)
**Prelabor rupture of the membranes < 37 weeks^**				
Median gestational age – wk°	32.0[a] (30.4-33.5)	35.9 (34.6-36.2)		-3.9 (-5.6 to 0.6)
Rupture at < 37 wk – no. (%)°	23/47 (49)	4/41 (10)	5.02 (2.05-13.2)	39 (23 to 60)
Rupture at < 34 wk – no. (%)°	18/47 (38)	1/41 (2)	15.7 (2.92-90.8)	36 (20 to 54)
**Gestational age at birth^**				
Median gestational age – wk°	34.6 (31.9-36.7)	38.4 (36.6-39.0)		-3.9 (-4.9 to -2.1)
Gestational age group – no. (%)				
<37 wk°	35/47 (74)	11/41 (27)	2.78 (1.70-4.85)	48 (31 to 70)
<34 wk°	19/47 (40)	0/41 (0)		40 (25 to 58)
<32 wk°	12/47 (26)	0/41 (0)		26 (10 to 41)
**Placental abruption** – no. (%)°	1/47 (2)	1/41 (2)	0.87 (0.09-8.21)	0 (-11 to 12)
**Neonatal outcomes in live births***				
Birthweight – gr°	2287 (1745-2537)	2816 (2490-3170)		-542 (-900 to -296)[a]
Neonatal repair of defect – no. (%)	23/45 (51)	15/41 (37)	1.40 (0.86-2.32)	15 (-6 to 37)
Use of patch – no. (%)	20/23 (87)	12/15 (80)	1.09 (0.81-1.62)	7 (-23 to 33)
Time to repair – days	2 (2-5)	7 (5-9)		-5 (-6 to -2)
ECMO – no. (%)	3/45 (7)	13/41 (32)	0.21 (0.07-0.63)	-25 (-43 to -8)

Data on intention-to-treat population unless specified otherwise. Results are reported as absolute numbers and percentages (categorical variables) or as medians and interquartile ranges (continuous variables). [#]difference between medians / percentages 95% confidence interval unless specified otherwise. °Exploratory outcomes. ^3 terminations of pregnancy excluded from the expectant management group; *2 cases of neonatal palliative care in the FETO group and 3 terminations of pregnancy in the expectant management group were excluded. [a]One (1%) missing value. The analysis addressed missing values as per-protocol (see Protocol and Statistical Analysis Plan).

Click to go to top of document: table of contents

Supplementary Table S8: Occurrence of adverse events in the safety population including overrunning patients

	FETO n = 47	Expectant n = 41^
**Serious adverse events**		
Fetal death within 24 hours after FETO	0/47 (0)	n.a.
Placental abruption within 24 hours after FETO	0/47 (0)	n.a.
Fetal death at any time in pregnancy	0/47 (0)	0/41 (0)
Placental abruption at any time in pregnancy	1/47 (2)	1/41 (2)
Placental laceration at balloon removal°	1/47 (2)	n.a.
Neonatal death due to failure of balloon removal¨	1/47 (2)	n.a.
Tracheomalacia†	1/47 (2)	0/41 (0)
Chorioamnionitis#	0/47 (0)	1/41 (2)
Increased biochemical inflammatory markers after PPROM#	1/47 (2)	0/41
Abnormal cardiotocography prelabor#	2/47 (4)	1/41 (2)
Decreased fetal movements leading to hospital admission#	1/47 (2)	1/41 (2)
Hospital admission due to preterm contractions, however delivery at term#	1/47 (2)	0/41 (0)
Preterm prelabor rupture of membranes <37 weeks	23/47 (49)	4/41 (10)
Delivery <37 weeks	35/47 (74)	11/41 (27)
Head retention during breech delivery#	1/47 (2)	0/41 (0)
Neonatal death <28 days	30/47 (64)	32/41 (78)
Death between 28 days and 6 months	0/47 (0)	3/41 (7)
Perinatal asphyxia (umbilical pH <7.00)	1/29 (3)	2/30 (7)
Extra Corporeal Membrane Oxygenation	3/47 (6)	13/41 (32)
Bronchopulmonary dysplasia in survivors to discharge	12/17 (71)	5/6 (83)
Pulmonary hypertension in survivors to discharge	16/17 (94)	6/6 (100)
Periventricular leukomalacia in survivors to discharge	1/17 (6)	0/6 (0)
Sepsis in survivors to discharge	10/17 (59)	6/6 (100%)
Necrotizing enterocolitis in survivors to discharge	0/13 (0)	0/6 (0)
Intranventricular hemorrhage in survivors to discharge	0/17 (0)	0/6 (0)
**Non-serious adverse events**		
Bleeding at trocar insertion during fetoscopy#	1/47 (2)	n.a.
Polyhydramnios first presenting at follow up US	12/41 (29)	-
Pregnancy induced hypertension#	0/47 (0)	1/41 (2)
Chorioamniotic membrane separation	9/43 (21)	-
Gastroesophageal reflux in survivors to discharge	11/15 (73)	3/6 (50)

All results are reported as absolute numbers and percentages. #Events reported via a free text field; others were check boxes (yes/no). For the latter, the denominator takes into account missing values. Abbreviations: n.a. = not applicable, - = not reported in the expectant management group.

The safety population used in here includes only randomized patients who effectively received the treatment they were allocated to (Section 2A of statistical analysis plan). When not specified, events are calculated in the full safety population. ^This group excludes three cases that underwent termination of pregnancy. °1 case with massive placental bleeding during trocar insertion, leading to emergency cesarean section and to neonatal death; ¨In 1 woman who was not compliant and moved away from the FETO center; the balloon could not be removed at her local unit; †1 infant was diagnosed at 10 months of age with tracheomalacia, but this child had previously undergone multiple surgeries.

Click to go to top of document: table of contents

Supplementary Table S9: Operative outcomes in patients in the FETO arm including overrunning patients

Characteristic	
**FETO procedure (n=47)**	
Gestational age at FETO – wk	28.6 (28.0 – 29.1)
Anesthesia: local – no. (%)	35/47 (74)
Anesthesia: neuraxial block – no. (%)	10/47 (21)
Anesthesia: general – no. (%)	2/47 (4)
**Balloon removal procedure (n=45*)**	
Gestational age at balloon removal – wk	34.0 (32.1 – 34.4)
Emergency balloon removal – no. (%)†	17/45 (38)[a]
Gestational age – wk	32.0 (31.0 – 33.1)[a]
Method: fetoscopy – no. (%)	6/17 (35)[a]
Method: ultrasound guided puncture – no. (%)	2/17 (12)
Postnatal – no. (%)	9/17 (53)
Elective balloon removal† – no. (%)	26/45 (58)[b]
Gestational age – wk	34.3 (34.0 – 34.6)[b]
Method: fetoscopy – no. (%)	25/26 (96)[b]
Method: ultrasound guided puncture – no. (%)	1/26 (4)
No removal because of spontaneous deflation - no. (%)	2/45 (4)
**Interval between balloon insertion and removal (n=45*)**	
Duration of tracheal occlusion – days	34 (28 – 41)[c]
Change in O/E LHR from baseline – %	59 (25 – 119)[d]
**Interval between balloon removal and delivery (n=47)**	
<24 hours - no. (%)	17/47 (36)

Continuous variables are reported as medians and interquartile ranges, categorical variables as absolute numbers and percentages. Abbreviations: FETO: fetoscopic endoluminal tracheal occlusion; GA: gestational age; O/E LHR: observed to expected lung-to-head ratio. All exploratory outcomes except for change in O/E LHR from baseline.

*In two cases balloon removal was not attempted at birth, because care was withdrawn. [a]Including one fetus where the balloon was deflated. [b]Including two fetuses where the balloon was deflated, [c]Excluding five spontaneous balloon deflations; [d]Five (11%) missing values. †Reasons given were: prelabor membrane rupture (n=10), preterm labor (n=6), fetal distress (n=1). Abbreviations: FETO: fetoscopic endoluminal tracheal occlusion.

Click to go to top of document: table of contents

Supplementary Table S10: Secondary neonatal outcomes in survivors to discharge including overrunning patients

Outcome	FETO (n=17)	Expectant (n=6)
Days to neonatal repair of defect	2 (2-5)	4 (1-8)
ECMO days in survivors receiving ECMO (n=3)	nc (n=1)[a]	nc (n=2)[a]
Days of ventilatory support	40 (23-90)	40 (18-53)
Days to full enteral feeding[b]	29 (19-42)	31 (18-46)
Bronchopulmonary dysplasia – no. (%)	12/17 (71)	5/6 (83)
Mild	4/12 (33)	2/5 (40)
Moderate	6/12 (50)	1/5 (20)
Severe	2/12 (17)	2/5 (40)
Pulmonary hypertension – no. (%)	16/17 (94)	6/6 (100)
Periventricular leukomalacia – no. (%)	1/17 (6)	0/6 (0)
Sepsis – no. (%)	10/17 (59)	6/6 (100)
Intraventricular hemorrhage > grade III – no. (%)	0/17 (0)	0/6 (0)
Retinopathy of prematurity > grade 3 – no. (%)	0/17(0)	0/6 (0)
Necrotizing enterocolitis – no. (%)[c] °	0/13 (0)	0/6 (0)
Gastroesophageal reflux – no. (%)[b]	11/15 (73)	3/6 (50)
NICU days	52 (36-105)	85 (74-107)

Results are reported as absolute numbers and percentages (categorical variables) or as medians and interquartile ranges (continuous variables). °Exploratory outcomes. [a]nc: not calculated because n too low or percentage of 0 in both arms. [b]Two (9%) missing values. . [c]Four (17%) missing values.

Click to go to top of document: table of contents

Supplementary Table S11: Interim analyses

						One-sided analysis[c]		Two-sided analysis[c]	
**Analysis stage**	**Date**	**Target N/arm**	**FETO, n (%)**	**Expectant management, n (%)**	**Difference between %**	**p-value**	**Alpha[d]**	**p-value**	**Alpha[d]**
Interim 1	Oct 17	23	11/24 (45.8)	3/22 (13.6)	32.2	0.0057	0.0005	0.0114	0.0010
Interim 2	Sep 19[a]	35	13/32 (40.6)	6/38 (15.8)	24.8	0.0077	0.0036	0.0154	0.0072
Interim 3	Mar 20	40[b]	16/40 (40.0)	6/40 (15.0)	25.0	0.0046	0.0064	0.0091	0.0128
Interim 3 (with overrunning patients)	Jan 21	na	17/47 (36.2)	6/44 (13.6)	22.5	0.0053	0.0109	0.0105	0.0218[e]
Interim 4	-	46	Not done						
Interim 5	-	52	Not done						
Final	-	58	Not done						

[a]One of the infants included in the second interim was hospitalized at NICU for a long time, making the primary outcome (survival at discharge) unknown. By the time this outcome was available, we had complete outcomes of the subsequent 10 cases, making already the third interim analysis possible as well. Meanwhile, the trial statistician indicated that the p-value would not meet the alpha whether or not this case survived to discharge.

[b]The new statistician implemented a group-sequential design with block randomization to have equal numbers in each arm at every interim analysis, which became effective after the second interim analysis.

[c]The statistical design and analysis was done using a one-sided test (while controlling the overall alpha at 2.5%) focusing on superiority of FETO. We have post hoc added the more conventional two-sided results (for which the overall alpha would be 5%), which equal twice the one-sided results if the observed effect favored FETO. A two-sided approach at an overall alpha of 5% is conceptually identical to a one-sided approach at an overall alpha of 2.5%, and would have led to exactly the same conclusions by definition. The difference is that a two-sided approach could also yield a low p-value if FETO would be inferior to expectant management, whereas the one-sided approach would not because it only evaluates superiority of FETO. Therefore, with a two-sided approach, the direction of the observed effect must be checked.

[d]The alpha level at each analysis stage is defined using the O'Brien-Fleming method in order to control the overall alpha at 2.5% (one-sided) or 5% (two-sided).

[e]Recalculated alpha level when replacing (i.e. ignoring) the original third interim analysis.

Observed survival rates for both study arms:

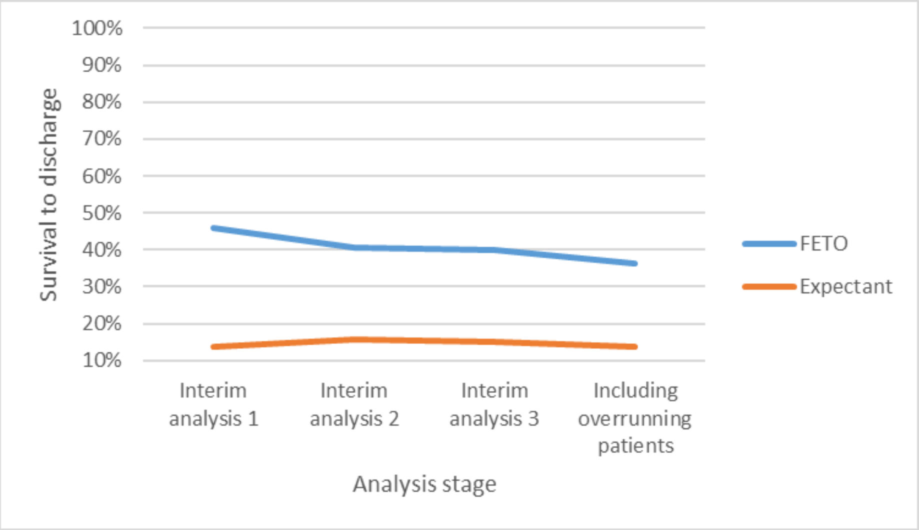

Click to go to top of document: table of contents

Supplementary Table S12: Case load for postnatal management centers two years prior to their first trial patient.

Center	Year 1	Year 2
1st Department of Obstetrics and Gynecology, Medical University of Warsaw	22	28
CHU Clermont-Ferrand	7	4
CHU Sainte Justine Université de Montréal	7	7
CHRU Lille	13	16
CHRU Montpellier	4	5
Erasmus MC, Rotterdam	11	14
HFME Lyon, Lyon	15	9
Hospital San Joan de Deu, Barcelona	14	14
Hôpital Bicêtre, Paris, France	17	13
Hôpital Robert Debré, Paris	8	8
Hôpital Trousseau, Paris	7	6
Hôpitaux de Toulouse Paule de Viguier, Toulouse	4	3
Japan National Center for Child Health and Development, Tokyo	9	9
King's College London	23	20
Ospedale Maggiore Policlinico, Milano	15	18
Ospedale Pediatrico Bambino Gesù, Rome	16	17
Radboudumc/Amalia Children's Hospital Nijmegen	6	22
St George's Hospital, London, United Kingdom	10	7
Saint Luc UCL, Brussels	4	2
Schiltigheim, Centre Medico-Chirurgical et Obstetrical, Strasbourg	4	7
SickKids Hospital, Toronto	21	34
Universitätsklinikum Bonn	14	11
University Hospitals Leuven	15	11
University Hospital Zurich	8	7
University of Texas Health Science Center/ Children's Memorial Hermann, Houston	11	3

Twelve postnatal centers did not provide historical data

Click to go to top of document: table of contents
